# Rethinking polyphenol oxidases in wheat: beyond the “low-PPO is always better” paradigm

**DOI:** 10.3389/fgene.2026.1810045

**Published:** 2026-07-15

**Authors:** Anna Paola Minervini, Silvia Procino, Anna Maria Pellegrini, Alberto Reddavide, Domenico De Paola, Giacomo Mangini, Nunzio D’Agostino, Francesca Taranto

**Affiliations:** 1 Institute of Bioscience and BioResources (CNR-IBBR), Bari, Italy; 2 Department of Agricultural Sciences, University of Naples Federico II, Naples, Italy

**Keywords:** allelic variants, biotic and abiotic stresses, browning, climate change, polyphenol oxidases, *Triticum*

## Abstract

Polyphenol oxidase (PPO) activity is a key determinant of wheat quality, influencing enzymatic browning of end products while also contributing to biochemical defence against (a)biotic stresses. In this review, we present a comprehensive structural characterization of more than 25 distinct alleles across the Ppo-1 and Ppo-2 gene families, revealing extensive allelic diversity between bread wheat (*Triticum aestivum* L.) and durum wheat (*Triticum turgidum* ssp. *durum (Desf.) Husn.*). Comparative analyses across the A, B, and D genomes reveal substantial structural polymorphisms that contribute to functional diversification of Ppo genes. Our synthesis indicates that this broad allelic repertoire provides a genetic toolkit for fine-tuning PPO activity across tissues and phenological stages. This regulation is important for environmental adaptation while maintaining high processing quality standards. Emerging evidence challenges the prevailing assumption that “low-PPO is always better.” Higher PPO activity levels in certain cultivars are often associated with more robust defence responses, potentially providing greater protection against pathogens and environmental stressors. However this relationship remains largely correlative and requires functional validation. These observations suggest that excessive suppression of PPO activity may inadvertently weaken plant resilience. Overall, the data support a paradigm shift toward strategic modulation of PPO activity, balancing the maintenance of plant defence across diverse agro-climatic environments with the preservation of end-use quality in wheat-derived products.

## 1 Introduction

Polyphenol oxidases (PPOs) are copper-containing enzymes widely distributed among higher plants, animals, and microorganisms (Zhang, 2023). Their absence in several chlorophytes (green algae) and in Arabidopsis species (*Arabidopsis lyrata* and *A. thaliana*) indicates that they are not required for primary metabolism but function in specialized processes associated with secondary metabolism (Tran et al., 2012). Although the browning reaction mediated by PPOs in damaged plant tissues upon exposure to oxygen is well known and extensively characterized, increasing evidence indicates that these enzymes are associated with plant responses to biotic and abiotic stresses (Tran et al., 2012; Wold-McGimsey et al., 2023).

Within the genus *Triticum*, PPOs were initially isolated and characterized in bread (*Triticum aestivum* L.) and durum wheat (*Triticum turgidum* ssp. *durum* (Desf.) Husn.) (Marsh and Galliard, 1986; Hatcher and Kruger, 1993; Kocaçaliskan et al., 1995; Pasqualone et al., 2001; Altunkaya and Gökmen, 2012), the two most economically important and widely cultivated wheat species (de Sousa et al., 2021). The structural diversity and diversification of Ppo gene families in bread and durum wheat directly mirror the genetic complexity arising from their distinct allopolyploid evolutionary histories. This process began with the convergence of diploid ancestors (2n = 2x = 14), where the hybridization between *Triticum urartu* (AA genome) and a species related to *Aegilops speltoides* (the presumed donor of the BB genome) produced the tetraploid wild emmer wheat, *T. turgidum* ssp. *dicoccoides* (2n = 4x = 28, BBAA) (Marcussen et al., 2014).

This tetraploid lineage served as the foundation for durum wheat, which emerged from the domestication of *T. turgidum* ssp. *dicoccum* (Maccaferri et al., 2019). In contrast, a second major evolutionary leap occurred when domesticated emmer hybridized with the diploid wild grass *Aegilops tauschii* (2n = 2x = 14, DD), ultimately giving rise to the allohexaploid bread wheat (*T. aestivum*, 2n = 6x = 42, BBAADD) (He et al., 2009; Wang et al., 2018; Levy and Feldman, 2022). Consequently, the Ppo gene family in bread wheat has a more complex architecture than that of durum wheat because of the additional contribution of the D-genome homoeologues.

These polyploidization events retained multiple Ppo-coding genes across the different subgenomes (homoeologues), which later underwent intra-chromosomal duplications to generate paralogous copies.

Over evolutionary time, these genes have continued to accumulate mutations within both cultivated wheats and their wild progenitors (Massa et al., 2007; Fuerst et al., 2008).

As with other crops, wheat breeding has more recently prioritized the enhancement of grain quality and technological performance by focusing on the reduction of PPO activity through the selection of favourable alleles (He et al., 2007; Wang et al., 2009; Morris, 2018). However, this primary objective has often been pursued without considering the potential beneficial roles that PPOs may play in stress adaptation and overall plant performance. Ppo genes exhibit a dual nature. Their structural complexity has historically complicated mapping, amplification and sequencing (Massa et al., 2007), yet this same complexity makes them a rich resource for studies in evolution, genome-wide association analyses, and genome editing.

This review provides a comprehensive and up-to-date overview of PPO enzymes in bread and durum wheat, with a particular emphasis on their genomic architecture and allelic diversity. We synthesize current knowledge on the identification, characterization, and expression of Ppo genes, highlighting both established methodologies and emerging strategies for modulating their activity. Moving beyond the conventional view that low PPO activity is the primary target in wheat breeding, we advocate for a more nuanced approach that integrates these enzymes into holistic improvement strategies. Although Ppo genes have proven valuable as molecular markers for reconstructing wheat evolutionary history, we seek to redefine their functional significance within a complex regulatory framework. Rather than viewing them simply as undesirable factors affecting end-product quality, we consider them key regulators of wheat responses to biotic and abiotic stresses. We propose that PPO activity can be modulated, rather than simply suppressed, by exploiting the plant bifurcated genetic system of constitutive and inducible expression (Sultan et al., 2025). In this context, PPOs act as dynamic components of the plant defence system: distinguishing between expression pathways may allow the maintenance of low grain PPO levels to preserve industrial quality, while enhancing localized, stress-induced activity in vegetative tissues. This perspective raises the possibility that intensive selection for low PPO activity may have inadvertently compromised plant performance, revealing trade-offs between reduced enzymatic browning and yield stability under variable environmental conditions. To reconcile biological imperatives with industrial requirements, we examine integrated mitigation strategies that combine genetic modulation with exogenous interventions (i.e., chemical treatments, physical methods, etc.). Drawing on insights from other crop species, we show how exploiting PPO functional diversity can strengthen defence mechanisms and improve environmental resilience. Overall, we argue that precise, stage-specific modulation of PPO activity offers a more effective strategy than complete genetic silencing for adapting wheat to a changing climate. We conclude by highlighting key knowledge gaps and proposing future research directions to better align industrial quality requirements with the biological demands of stress adaptation.

### 1.1 PPO activity in wheat: implications for quality and processing

**FIGURE 1:**
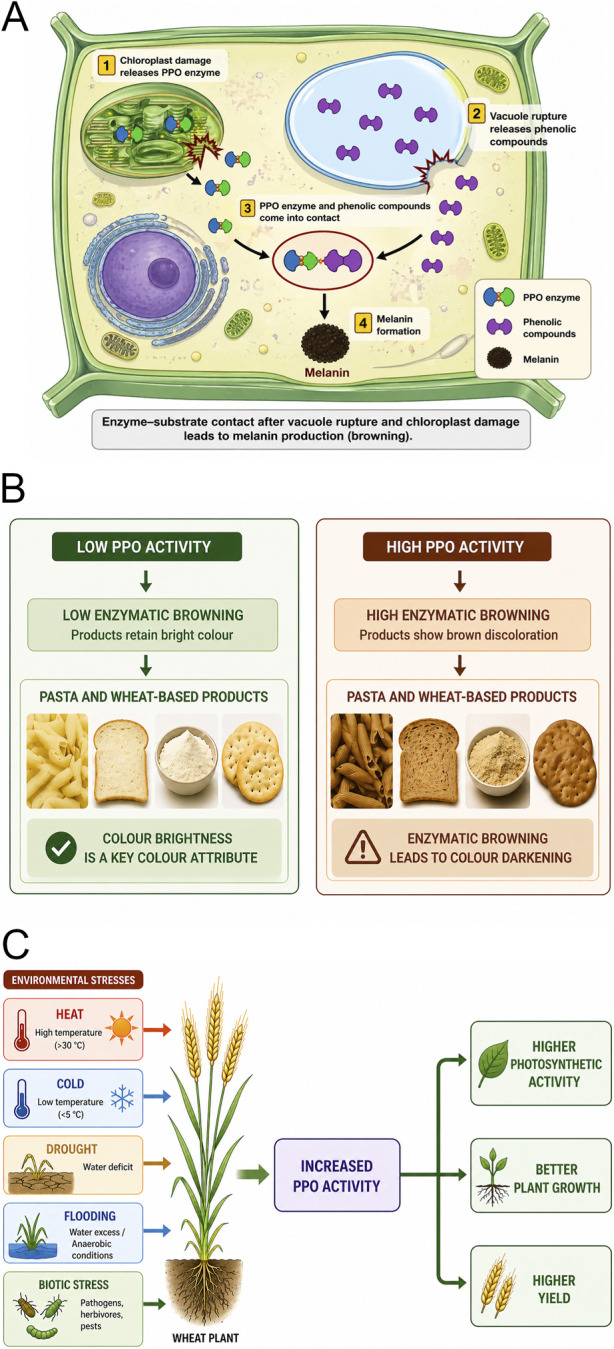
Role of polyphenol oxidases (PPOs) in wheat grain quality and stress responses. **(A)** In intact cells, PPO enzymes are compartmentalized in chloroplasts, while phenolic substrates are stored in vacuoles. Tissue damage disrupts these compartments, allowing enzyme–substrate interaction and leading to the formation of dark melanins, which strongly influence wheat technological performance and the visual quality of processed products. **(B)** PPO activity and grain quality: Low PPO activity is essential to maintain bright colour in pasta and other wheat-based products, whereas high PPO activity promotes enzymatic browning and undesirable darkening during processing. **(C)** PPO activity and stress responses: Abiotic (e.g., heat, drought, flooding). and biotic stresses modulate PPO activity, with downstream effects on plant physiology, including photosynthetic performance, growth, and ultimately yield. Image generated with ChatGPT Edu (OpenAI).

PPOs are copper-containing metalloproteins characterized by two highly conserved copper-binding domains, CuA and CuB, each coordinated by three histidine residues (Aydin et al., 2015). The well-defined active site of the enzyme binds copper ions. It enables interactions with molecular oxygen and phenolic substrates, thereby catalysing the oxidation of phenolic compounds into unstable o-quinones (Palma-Orozco et al., 2011). These highly reactive intermediates subsequently undergo spontaneous secondary reactions, leading to the formation of complex polymers such as black- or brown-coloured melanins (Steffens et al., 1994; Rolff et al., 2011) (Figure 1A).

The synthesis of o-quinones proceeds through two oxygen-dependent reactions: (i) the hydroxylation of monophenols to *o*-diphenols, catalysed by monophenol oxidase (cresolase, or tyrosinase activity, EC 1.14.18.1) and (ii) the subsequent oxidation (dehydrogenation) of *o*-dihydroxyphenols to *o*-quinones mediated by diphenol oxidase or catecholase activity (Vámos-Vigyázó and Haard, 1981; Steffens et al., 1994; Yoruk and Marshall, 2003; Taranto et al., 2017).

In wheat, PPOs are predominantly localized in the bran fraction of the grains, with only trace levels detected in the germ (Marsh and Galliard, 1986; Hatcher and Kruger, 1993; Okot-Kotber et al., 2001). These enzymes are intracellular and primarily associated with chloroplasts, specifically within the thylakoid lumen, whereas their phenolic substrates are sequestered in the vacuoles (Mayer and Harel, 1979; Vaughn and Duke, 1984). Due to this strict compartmentalization, PPO activity necessitates the physical disruption of cellular integrity (e.g., tissue wounding) to facilitate enzyme-substrate contact (Boeckx et al., 2015). Purified wheat PPOs exhibit a high affinity for *o*-diphenolic and triphenolic substrates, including catechol, caffeic acid, gallic acid and pyrogallol, and exhibit optimal activity at approximately pH 6.5 and 35 °C (Altunkaya and Gökmen, 2012; Yu et al., 2024).

Dough formation represents a critical processing stage during which enzymes and substrates come into close contact, thereby increasing susceptibility to enzymatic browning (Feillet et al., 2000). Consistent differences in PPO activity have been reported between bread and durum wheat, with the latter generally exhibiting lower PPO activity (Demeke and Morris, 2002).

This characteristic is considered advantageous for end-use quality, particularly in products such as semolina and pasta, where colour brightness is a key quality attribute (Figure 1B). The reduced PPO activity observed in durum wheat has been attributed to genetic factors, including allelic variants associated with lower Ppo expression, as well as to targeted breeding efforts aimed at improving product colour (Simeone et al., 2002; Mangini et al., 2014). The impact of PPO activity in wheat and wheat-derived products is influenced by both grain maturity and processing conditions. In food production, thermal treatments effectively inactivate PPOs; consequently, dry or pasteurized products are less susceptible to enzymatic browning than fresh products such as noodles or pasta (Taranto et al., 2012). Accordingly, the food industry has largely focused on minimizing PPO levels in wheat cultivars (Nakamaru et al., 2023), as PPO activity in mature grains is associated with pigment accumulation and 'black point' discoloration, which adversely affects overall wheat quality (Liu et al., 2017). Nevertheless, PPO-mediated oxidation may also play a beneficial role in grain preservation by enhancing resistance of dormant seeds to decay (Fuerst et al., 2014). Breeding efforts aimed at genetically reducing PPO activity are therefore aligned with the growing consumer demand for minimally processed foods (Moon et al., 2020). At the same time, consumer studies indicate a greater tolerance for colour imperfections than for defects related to shape or other physical attributes, highlighting the need to balance aesthetic quality with functional and agronomic traits (Lagerkvist et al., 2023).

### 1.2 PPO in plant physiology and stress responses

Although primarily recognized for their detrimental role in postharvest browning, PPOs also function as key physiological markers and integral components of the plant defence system against a wide range of environmental challenges. Extensive genomic and proteomic evidence indicates that Ppo gene expression and PPO catalytic activity are markedly induced in response to both biotic and abiotic stressors, including herbivores, pathogen infection, drought, cold, salinity, and mechanical injury (Figure 1C) (Tuladhar et al., 2021; Hajam et al., 2023; Zhang, 2023). Increased PPO activity has been reported following infection or infestation by the plant-parasitic nematode *Pratylenchus thornei* (Rahaman et al., 2020), the aphid *Rhopalosiphum padi* L. (Xu et al., 2021), and several phytopathogenic fungi, including *Fusarium graminearum* (Mohammadi and Kazemi, 2002)*, Bipolaris sorokiniana* (Kumari et al., 2023; Chakraborty et al., 2025a) and *Puccinia striiformis f. sp. tritici* (El-Sharkawy et al., 2024). While these observations support a potential involvement of PPOs in stress responses, they do not establish a direct causal role in resistance.

Recent evidence suggests that PPOs may contribute to plant resistance through multiple interconnected mechanisms. PPO-mediated oxidation of phenolic compounds generates quinones and reactive oxygen species (ROS), which may exert direct toxicity toward insects and pathogens, promote oxidative damage in microbial cells, and induce alkylation of amino, phenolic, and mercapto groups in proteins, thereby reducing protein bioavailability and nutritional quality for herbivores and microorganisms (Zhang and Sun, 2021; Zhang, 2023). Mechanistically, PPO-mediated defence is primarily explained by a quinone-based model in which PPOs catalyze the oxidation of phenolic substrates into highly reactive quinones capable of impairing pathogen growth and survival. Quinones may also cross-link proteins and cell wall components, thereby reinforcing structural barriers against infection and reducing nutrient availability for invading organisms. Collectively, these findings support the hypothesis that PPOs may function not only as stress-associated markers, but also as active contributors to plant defence responses, although definitive mechanistic evidence remains limited in many species (Li and Steffens, 2002; Constabel and Barbehenn, 2008). Beyond these classical defence mechanisms, PPOs also appear to participate in broader stress-responsive signalling networks. Increasing evidence suggests that PPOs may function as downstream components of JA-dependent defence pathways involved in anti-herbivore responses and systemic resistance (Constabel and Ryan, 1998; Constabel et al., 2000). Experimental studies further support this association. In *Camellia sinensis*, feeding by *Ectropis grisescens* significantly increased JA accumulation, PPO activity, and CsPpo gene expression (Zhang et al., 2020). Moreover, exogenous methyl jasmonate treatment enhanced PPO activity, whereas inhibition of JA biosynthesis suppressed Ppo induction, supporting the role of JA as an upstream regulator of PPO-mediated defence responses.

Together, these findings suggest that PPOs may contribute to herbivore resistance as downstream components of JA signalling pathways. However, the precise toxic or anti-nutritional effects of PPO-derived quinones on insects remain incompletely understood.

PPO activity has also been associated with abiotic stress responses, particularly under drought and salinity. Elevated PPO activity under water deficit conditions has been linked to stress-induced alterations in phenolic metabolism, oxidative stress responses, and tissue senescence, although the magnitude of induction appears highly genotype dependent (Abdi et al., 2021). Arbuscular mycorrhizal symbiosis can mitigate drought-induced Ppo activation, indicating that Ppo regulation is integrated into broader physiological and symbiotic mechanisms of stress adaptation (Abdi et al., 2021). Similarly, Tanbakuyi et al. (2025) reported increased PPO activity in multiple wheat tissues under drought, with stronger induction generally observed in drought-sensitive genotypes. These findings suggest that Ppo upregulation under drought may primarily reflect stress intensity and genotype-specific metabolic adjustments rather than directly determining drought tolerance. Under saline conditions, PPO activity in wheat can also increase and may be further modulated by hormonal signalling pathways, such as strigolactones (Jariani et al., 2025). However, contrasting responses have also been reported. In wheat seedlings, PPO activity remained largely unchanged under salt stress alone but decreased significantly following treatment with osmoprotectants such as trehalose or mannitol were applied under saline conditions. This pattern contrasts with the generally increased activities of other antioxidant enzymes, such as Ascorbate Peroxidases (APXs) and Catalases (CATs), under similar treatments (Alhudhaibi et al., 2024).

These findings indicate that Ppo responses to salinity are complex and depend on interactions among stress intensity, exogenous compounds, and the broader antioxidant network.

Studies in plant species beyond wheat have suggested a potential link between PPO activity and the regulation of photosynthesis (Chen et al., 2018; Szymborska-Sandhu et al., 2020). Moreover, PPO activity appears to be closely associated with cellular redox homeostasis. PPO-mediated oxidation of phenolic compounds contributes to ROS generation while interacting with antioxidant defence systems, including superoxide dismutases (SOD), CATs, and APXs (Zhang et al., 2021; Hasanuzzaman et al., 2020; Rao et al., 2025). The concomitant increase in PPO and SOD activities frequently observed under stress conditions supports the hypothesis that PPOs may participate in oxidative stress acclimation and ROS homeostasis, although direct evidence for a ROS-scavenging role remains limited.

PPOs have also been proposed to influence phenylpropanoid metabolism by modulating the availability of phenolic substrates required for the biosynthesis of lignin, flavonoids, and other secondary metabolites, thereby indirectly contributing to lignification and cell wall strengthening (Sullivan, 2015).

Recent studies further suggest that PPOs are associated with antioxidant defence responses and hormone-mediated signalling during plant stress. In potato cultivars infected with *Erwinia carotovora*, increased PPO activity and gene expression were correlated with enhanced resistance, elevated phenolic accumulation, and activation of antioxidant enzymes. In addition, salicylic acid (SA) and methyl jasmonate (MeJA) treatments significantly enhanced Ppo expression and activity, supporting the hypothesis that PPOs may participate in SA- and JA-dependent defence pathways and contribute to oxidative stress acclimation (Sobhy et al., 2024). However, these findings remain largely correlative, and functional validation through genetic and biochemical approaches is still scarce. Increasing evidence also indicates that Ppo regulation is highly tissue specific and developmentally controlled. In *Solanum melongena*, SmPPO10 is predominantly expressed in ripening fruit peel, where it contributes to defence against necrotrophic fungi, while remaining largely inactive in roots and seeds (Maioli et al., 2020). In *Salvia miltiorrhiza*, SmPPO1 and SmPPO4 are preferentially expressed in young roots, with SmPPO1 showing a 15-fold increase under methyl jasmonate treatment, whereas SmPPO11 is predominantly expressed in mature leaves (Li et al., 2017). Similarly, in globe artichoke, PPO1 and PPO2 exhibit tissue-specific expression patterns within leaves and floral organs (Pompili et al., 2023). Genome-wide analysis of *Populus trichocarpa* further suggest that Ppo genes contribute not only to defence but also to plant growth and organ development, with several PtrPPO genes highly expressed in actively growing tissues such as expanding leaves, roots, and shoot apices (He et al., 2021). Given the structural complexity and functional diversity of the Ppo gene family, precise modulation of their expression requires detailed characterization. Distinguishing Ppo isoforms induced by biotic stress in vegetative tissues from those regulating enzymatic activity in grains is essential. In cereal grains, Ppo induction promotes the formation of quinones and melanin, which contribute not only to browning but also to defensive cross-linking reactions and reinforcement of physical barriers against pathogens. A deeper understanding of these tissue-specific dynamics is therefore essential for moving beyond the 'low-PPO' selection bias, enabling the strategic fine-tuning of PPO activity to enhance crop resilience while preserving grain quality for industrial use and maintaining overall plant performance under increasing climate pressures.

### 1.3 Wheat PPOs: genetic insights

Given the significant role of PPOs in enzymatic browning, elucidating their genetic regulation and developing wheat cultivars with reduced PPO activity have long been central goals of breeding programmes (Baik et al., 1995; Demeke and Morris, 2002; Nilthong et al., 2012; Taranto et al., 2017; Morris, 2018; Siah and Quail, 2018; Zhai et al., 2020; Liu et al., 2020). Advances in understanding the genetic basis of PPO activity are therefore critical for improving wheat quality while maintaining agronomic performance. In wheat, the main characterized PPOs form a multigene superfamily comprising homoeologous and paralogous genes on the group 2 chromosomes that have originated through duplication and mutation events (Massa et al., 2007; Fuerst et al., 2008; Beecher and Skinner, 2011; Martin et al., 2011).

**FIGURE 2:**
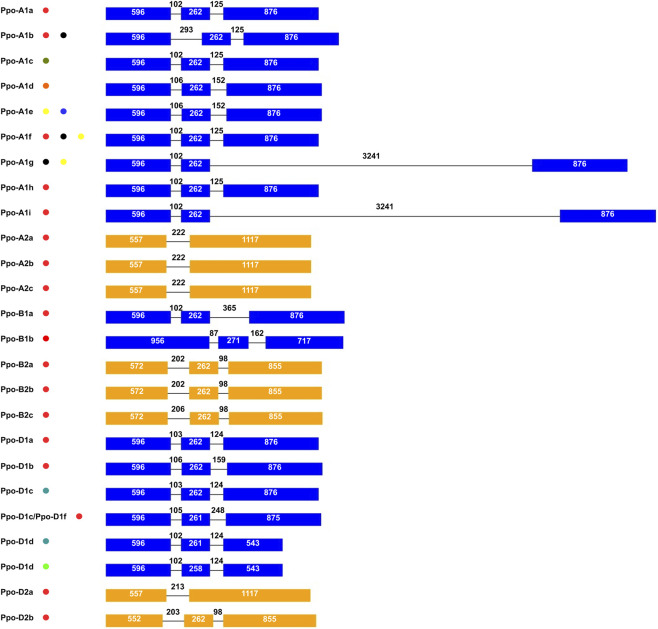
Structural architecture and allelic diversity of Ppo-1 and Ppo-2 genes across different wheat species and their wild relatives. Ppo-1 (blue) and Ppo-2 (orange) genes are shown with exons represented as coloured blocks and introns as horizontal lines, with lengths indicated in base pairs (bp). Species distribution is indicated by colour-coded circles adjacent to each locus, linking specific alleles to their respective taxa; *Triticum aestivum* (red), *Triticum turgidum* ssp. *dicoccoides* (yellow), *Triticum turgidum* ssp. *durum* (black), T. *urartu* (olive green), T. *boeoticum* (brown), T. *monococcum* (blue), *Ae. tauschii* (cyan), *Ae*. *tauschii* KT120-013 (bright green).

The first gene family, Ppo-1, comprises the copies Ppo-A1 and Ppo-D1 (Beecher and Skinner, 2011), which are located on the long arms of the group 2 chromosomes (Figure 2, s1; Table 2).

The second gene family, Ppo-2, includes the homoeologous copies Ppo-A2, Ppo-B2, and Ppo-D2 (Massa et al., 2007; Beecher and Skinner, 2011; Beecher et al., 2012; Taranto et al., 2015), also located on group 2 chromosomes, approximately 10 cM apart and closer to the long-arm telomeres (Beecher et al., 2012; Taranto et al., 2015).

The major genes and allelic variants of the Ppo family are summarized in Table 2 and Figure 2.

### 1.4 Ppo-A1, the major Ppo gene.

Early studies using Chinese spring substitution lines (i.e., genetic stocks in which a single pair of chromosomes is replaced by a homologous pair from a donor variety) consistently linked PPO activity to genes on chromosomes of homoeologous group 2, with chromosome 2A identified as the primary contributor (Jimenez and Dubcovsky, 1999). Subsequent mapping in bread wheat populations confirmed a major Quantitative Trait Locus (QTL) on the long arm of chromosome 2A, accounting for 37.9%–50.0% of the phenotypic variance in PPO activity (Demeke et al., 2001; Watanabe et al., 2004; Zhang et al., 2005; Raman et al., 2005). Similarly, in tetraploid durum wheat, (Simeone et al., 2002) identified a high-PPO QTL on the long arm of chromosome 2A, strongly associated with the RFLP marker *Xutv1427-2A*.

In *Triticum*, the Ppo-A1 gene consists of three exons (Demeke and Morris, 2002; Sun et al., 2005) (Table 2; Figure 2). All Ppo-A1 alleles share a1,731 bp open reading frame (ORF) encoding a of 577-amino-acid polypeptide.

(https://plants.ensembl.org/Triticum_aestivum/Gene/Summary?g=TraesCS2A02G468200;r=2A:712187112-712189567;t=TraesCS2A02G468200.1;db=core). To identify Ppo-A1 gene across various *Triticum* species, the PPO18 marker is frequently utilized as a prominent STS marker designed to amplify a specific diagnostic fragment (Sun et al., 2005; He et al., 2007; 2009; Taranto et al., 2012).

In *T*. *aestivum* genetic maps, it is genetically linked to the Simple Sequence Repeat (SSR) markers *Xgwm312* and *Xgwm294* at distances of 1.4 cM and 5.8 cM, respectively (Röder et al., 1998; Sun et al., 2005). The Ppo-A1 gene (TraesCS2A02G468200.1) exhibits five well-characterized allelic variants: Ppo-A1a, Ppo-A1b, Ppo-A1f, Ppo-A1h and Ppo-A1i (Sun et al., 2005; He et al., 2007; 2009; Beecher et al., 2012) (Table 1). PPO18 marker distinguishes between different alleles based on a 191-bp insertion located within the first intron. The insertion produces an 876-bp fragment associated with low PPO activity (allele Ppo-A1b), while its absence yields a 685-bp fragment linked to high PPO activity (allele Ppo-A1a) (Sun et al., 2005). Additionally, two-point mutations at positions 341 (C→G) and 788 (G→A) have been observed in the amplified DNA sequence from low-PPO cultivars (Sun et al., 2005). In addition, alleles Ppo-A1e and Ppo-A1g are typically associated with cultivars exhibiting moderate/low PPO activity, whereas Ppo-A1f correlates with high PPO activity (Taranto et al., 2012; Mangini et al., 2014; Harisha et al., 2023). Additional polymorphic sequences have been identified in related species: Ppo-A1c in *T. urartu*, Ppo-A1d (putative) in *T. boeoticum*, Ppo-A1e (putative) in *T. monococcum* and *T. turgidum* ssp. *durum,* Ppo-A1f in *T. turgidum* ssp. *dicoccoides* and Ppo-A1g in *T. turgidum* ssp. *durum* (He et al., 2009). The first introns of all Ppo-A1 alleles conform to the canonical GT–AG splice rule and exhibit limited sequence variability. In contrast, the second introns possess a GC donor site and a canonical AG acceptor site and display a higher frequency of single nucleotide polymorphisms (SNPs), insertions–deletions (InDels) and miniature inverted-repeat transposable elements (MITEs). Generally, shorter insertions within these introns are associated with reduced PPO activity (Massa et al., 2007; He et al., 2009). In the durum wheat cultivar Svevo, the Ppo-A1 gene (locus TRITD2Av1G261300, Rel. 1.0) exhibits four distinct splicing variants or isoforms. Among these, transcript version 0.2 is designated as the canonical sequence, encoding a primary protein of 577 amino acids (https://plants.ensembl.org/Triticum_turgidum/Gene/Compara_Ortholog?g=TRITD2Av1G261300;r=2A:706301918-706306994).

**TABLE 1:** Overview of the polyphenol oxidase (PPO) gene superfamily on homoeologous group 2 across various *Triticum* species, showing gene IDs, standardized nomenclature and chromosomal locations.

Species	Gene ID	Gene name	Chromosome
*T*. *aestivum*	TraesCS2A03G1101100*	Ppo-A1	2A
	TraesCS2A03G1101800*	Ppo-A2	2A
	TraesCS2B02G491000*	Ppo-B2	2B
	TraesCS2B03G1236300*	SSPPO-B1	2B
	TraesCS2D03G1045900*	Ppo-D1	2D
	TraesCS2D03G1047900*	Ppo-D2	2D
	TraesCS2A02G468200**	Ppo-A1	2A
	TraesCS2A02G468500**	Ppo-A2	2A
	TraesCS2B02G491100**	SSPPO-B1	2B
	TraesCS2B02G491400**	Ppo-B2	2B
	TraesCS2D02G468200**	Ppo-D1	2D
	TraesCS2D02G468600**	Ppo-D2	2D
*T. turgidum* ssp. *durum*
	TRITD2Av1G261300.2	Ppo-A1	2A
	TRITD2Av1G261390.1	Ppo-A2	2A
	TRITD2Bv1G224170	Ppo-B2	2B
*T. turgidum* ssp*. dicoccoides*
	TRIDC2AG066350	Ppo-A1	2A
*T. urartu*
	TuG1812G0200005170.01	Ppo-A1	2
*T. spelta*
	TraesTSP2B01G531200	Ppo-B	2B
	TraesTSP2A01G502400	Ppo-A	2A
	TraesTSP2D01G509700	Ppo-D	2D
	TraesTSP2B01G531300	Ppo-B	2B

*IWGSC CS, Refseq2.1 (GeneBank Assembly = GCA_018294505.1).

**IWGSC CS, Refseq1.1 (GeneBank Assembly = GCA_900519105.1).

Studies using the Svevo reference genome (Release 1.0) showed that the Ppo-A1g allele (TRIDC2AG066350) - found in durum and emmer wheat - features approximately 3 kb insertion in the second intron. Furthermore, recent findings indicated that the Ppo-A1g allele of tetraploid wheat is the progenitor of Ppo-A1i in some lines of bread wheat (Nakamaru et al., 2023).

Several additional primers have been developed for specific purposes. The PPO33 primer amplifies shorter fragments (290 bp and 481 bp) and specifically targets the 191 bp insertion in Ppo-A1 (He et al., 2007). The PPO33 primer has been applied in screening collections of wheat genotypes exhibiting both terminal heat tolerance and resistance to foliar diseases, facilitating the identification of desirable Ppo-A1 alleles for breeding programmes (Kumar et al., 2024).

The sequence of Ppo-A1g, from the *T. durum* cultivar Langdon, could not be fully amplified, likely due to multiple downstream mutations that interfere with PCR amplification failure and potentially lead to allele inactivation (He et al., 2009). To discriminate the Ppo-A1g allele, Mangini et al. (2014) developed the primer set MG18, which amplifies 493 bp for Ppo-A1b, 333 bp for Ppo-A1e, 302 bp for Ppo-A1f, or produces no PCR fragment for Ppo-A1g, all targeting the intron I sequence.

The Ppo-A1i allele is classified as a null allele, as no amplification was obtained using the PPO18 marker, and RNA-sequencing analysis failed to detect any transcripts (Hystad et al., 2015). However, Nakamaru et al. (2023) successfully amplified this allele using the PPO18 marker (He et al., 2007), which targets only the first exon, in combination with TaKaRa LA Taq® polymerase (TaKaRa) designed for long-range PCR. This approach produced a fragment of approximately 4 kb, revealing a 3,125 bp insertion, providing insight into the structural basis of the Ppo-A1i allele.

### 1.5 Ppo-B1, the least characterized Ppo gene

The gene Ppo-B1 remains the least characterized gene within the Ppo-1 locus. While the SSPPO-B1 gene (GenBank ID = AB254804, TraesCS2B03G1236300) is characterized and annotated in bread wheat, high-confidence gene models are still lacking in durum wheat genomic assemblies, despite the presence of some annotated sequences on public repositories (i.e., GenBank). This lack of robust annotation likely reflects the relatively minor contribution of Ppo-B1 to total PPO activity compared with its homoeologues. Consequently, its functional role remains incompletely resolved. Initial evidence for the existence of Ppo-B1 was provided by two independent studies, which reported a partial (GenBank ID = DQ889708) and a complete (GenBank ID = GQ303713) candidate gene (Massa et al., 2007; https://www.ncbi.nlm.nih.gov/nuccore/GQ303713) (Table 2).

**TABLE 2:** Allelic variation of characterized Ppo genes in *Triticum* and *Aegilops* species, including corresponding PPO activity, diagnostic Sequence-Tagged Site (STS) markers, GenBank accession numbers, and cited references.

Species	Allele	GenBank ID	PPO activity	STS markers	References
*Ae. Tauschii*	Ppo-D1c	EU371656	Low	PPO16	He et al. (2009)
*Ae. Tauschii*	Ppo-D1d	EU371657	Low	PPO16	He et al. (2009)
*Ae. Tauschii*	Ppo-D1d	LC843433	Low	PPO-D1d_F1/PPO-D1dR1	Nakamaru et al. (2025)
*T. boeoticum*	Ppo-A1d*	EU371652	High (putative)	PPO18	He et al. (2009)
*T. dicoccoides*	Ppo-A1b	EF070148	Low	n.a.	He et al. (2009)
*T. dicoccoides*	Ppo-A1f	EU371654	High	PPO18	He et al. (2009)
*T. durum*	Ppo-A1e	EU371653	High	PPO18	He et al. (2009)
*T. durum*	Ppo-A1f	n.a.	High	MG18	Mangini et al. (2014)
*T. durum*	Ppo-A1g	EU371655	Low	PPO18	He et al., 2009; Taranto et al., 2012
*T. monococcum*	Ppo-A1e*	EU371653	High (putative)	PPO18	He et al. (2009)
*T. urartu*	Ppo-A1c*	EU371651	High (putative)	PPO18	He et al. (2009)
*T .aestivum*	Ppo-A1a	EF070147	High	PPO18; PPO33; *Xgwn321*-2a; *Xgwm294*-2a; PPO18Plus	He et al. (2009)
*T. aestivum*	Ppo-A1b	EF070148	Low	PPO18	He et al. (2009)
*T. aestivum*	Ppo-A1f	EU371654	High	PPO18	He et al. (2009)
*T. aestivum*	Ppo-A1h*	JN632506	High (putative)	SNP assay + fluorescent labeling	Beecher et al. (2012)
*T. aestivum*	Ppo-A1i	LC770043	Inactive	PPO30; PPO18Plus	Nakamaru et al. (2023)
*T. aestivum*	Ppo-A2a	HQ228148	High	By cloning	Beecher and Skinner (2011)
*T. aestivum*	Ppo-A2b	HQ228149	Low	By cloning	Beecher and Skinner (2011)
*T. aestivum*	Ppo-A2c*	JN632507	Low (putative)	SNP assay + fluorescent labeling	Beecher et al. (2012)
*T. aestivum*	Ppo-B1a	GQ303713	Low	F-8	Si et al. (2012)
*T. aestivum*	Ppo-B1b	AB254804	Low	F-8	Si et al. (2012)
*T. aestivum*	Ppo-B2a	HQ228150	High	By cloning	Beecher and Skinner (2011)
*T. aestivum*	Ppo-B2b	HQ228151	Low	By cloning	Beecher and Skinner (2011)
*T. aestivum*	Ppo-B2c	JN632508	Low	SNP assay + fluorescent labeling	Beecher et al. (2012)
*T. aestivum*	Ppo-D1a	EF070149	Low	PPO16; STS01	He et al. (2007)
*T. aestivum*	Ppo-D1b	EF070150	High	PPO29	He et al. (2007)
*T. aestivum*	Ppo-D1c/Ppo-D1f	KJ567059	Inactive	CAPS PPOD1CAP	Hystad et al. (2015)
*T. aestivum*	Ppo-D2a	HQ228152	Low	By cloning	Beecher and Skinner (2011)
*T. aestivum*	Ppo-D2b	HQ228153	High	By cloning	Beecher and Skinner (2011)

n.a., not available; * indicates a putative allele for which functional validation is currently unavailable.

In contrast, Beecher et al. (2012) were unable to isolate the genes despite employing degenerate primer sets. Subsequently, Si et al. (2012) developed the STS primer pair F-8, which successfully amplified Ppo-A1, Ppo-B1 and Ppo-D1, enabling discrimination between the previously described alleles Ppo-B1a (GenBank ID = GQ303713) and Ppo-B1b (GenBank ID = AB254804). This assay was later applied to a limited set of Korean landraces for commercial evaluation, revealing a higher prevalence of the Ppo-B1b allele (Lee et al., 2025). The most comprehensive characterization of Ppo-B1 to date was reported by Taranto et al. (2015), who performed an in-depth analysis of Ppo-B1 and its neighbouring homoeologous Ppo-B2 in durum wheat. In this study, Ppo-B1 was mapped to the long arm of chromosome 2B (2BL), located 11.4 cM from Ppo-B2 and in proximity to the Diversity Arrays Technology (DArT) marker wPt-1140. The authors also developed a gene-specific functional marker, MG08, facilitating more precise allele discrimination. In addition to confirming the previously described alleles Ppo-B1a and Ppo-B1b, two new alleles, Ppo-B1c and Ppo-B1d, were identified. Structurally, Ppo-B1 closely resembles Ppo-A1 and Ppo-D1. However, intron II contains non-canonical GC-AG splice-sites and is approximately 200–240 bp longer (Si et al., 2012; Taranto et al., 2015).

Allelic variation is primarily concentrated within this second intron; notably, Ppo-B1c, the largest allele, was the only variant associated with elevated PPO activity (Taranto et al., 2015).

These findings suggest that Ppo-B1 expression may be modulated not only through alternative splicing but also by the presence of functional transcription factor-binding sites (TFBSs). Finally, recent genomic analyses of the Chinese spring reference genome have revealed the presence of two Ppo-1 genes on chromosome 2B (Ppo-1-B1 and Ppo-1-B2), likely arising from a duplication event (Liu et al., 2020; Wold-McGimsey et al., 2023), further highlighting the structural and evolutionary complexity of the Ppo gene family in wheat. However, recent genome sequencing of *T. turgidum* ssp. *durum* and *dicoccoides* has not annotated the Ppo-B1 gene sequence, which therefore remains to be identified and characterized.

### 1.6 Ppo-D1: the second most studied Ppo gene

The presence of a QTL associated with PPO activity on the long arm of chromosome 2D has been supported by multiple independent studies (Jimenez and Dubcovsky, 1999; Mares and Campbell, 2001; Raman et al., 2005). Building on this evidence, He et al. (2007) identified *in silico* and cloned Ppo-D1, revealing a gene structure comprising two introns, with the second intron flanked by non-canonical GC-AG splice sites, mirroring the structural organization observed in Ppo-A1. The same study developed two STS markers, PPO16 and PPO29, which enable discrimination between two allelic variants associated with high (Ppo-D1b) and low (Ppo-D1a) PPO activity, respectively. In addition, two primer sets (PPO3 and PPO4) were designed for full-length gene amplification (Supplementary Table S1). The STS marker PPO-05 amplifies both Ppo-A1 and Ppo-D1, producing a double PCR fragment in low-PPO cultivars, while STS01 specifically distinguishes between the Ppo-D1a and Ppo-D1b alleles (Wang et al., 2009).

Reduced enzymatic activity associated with the Ppo-D1 allele has been linked to two non-conservative amino acid substitutions in the encoded protein (Beecher et al., 2012). Consistent results were obtained by Chang et al. (2007), who developed the primer pair WP3-2 to amplify the second half of the Ppo-D1 gene. The close evolutionary relationship between Ppo-A1 and Ppo-D1 is further reflected in their identical ORF length of 1,731 bp, encoding a polypeptide of 577 amino acid residues. Additional allelic diversity at the Ppo-D1 locus was uncovered by He et al. (2009) through the identification of two novel variants in *Ae*. *tauschii*, designated Ppo-D1c and Ppo-D1d. While Ppo-D1c exhibits high sequence conservation, differing from Ppo-D1a by a single SNP, Ppo-D1d carries a 73 bp deletion in the third exon, which introduces a premature stop codon, resulting in a truncated protein of 466 amino acids. Both alleles are likely associated with low PPO activity, consistent with the moderate-to-low PPO levels reported in *Ae. tauschii* accessions (Chang et al., 2007; Fuerst et al., 2008; He et al., 2009). More recently, Nakamaru et al. (2025) demonstrated that the Ppo-D1d allele, which also harbours an additional 3 kb deletion in the 3′ untranslated region (UTR), is also present in the bread wheat line Fukuhonoka-NIL. The authors developed a co-dominant PCR-based marker spanning the region from the second intron to the end of the 73 bp deletion, enabling clear discrimination among the Ppo-D1a, Ppo-D1b, and Ppo-D1d alleles via standard agarose gel electrophoresis. Transcript analyses further confirmed that Ppo-D1d produces either a markedly reduced amount or undetectable mRNA levels (Nakamaru et al., 2025).

Several reports mention the existence of an additional putative allele, Ppo-D1e, although its structural polymorphisms remain uncharacterized and its occurrence within *Triticum* species is currently unknown (He et al., 2009; Hystad et al., 2015; https://graingenes.org/cgi-bin/GG3/report.cgi?class=allele;name=Ppo-D1e+(Triticum);id=9308). The most recently described null allele, Ppo-D1f, was identified in the *T. aestivum* line 07OR1074, which exhibits minimal PPO activity. This allele differs from Ppo-D1b by a single SNP, which was exploited to develop a cleaved amplified polymorphic sequence (CAPS) marker, PPOD1CAP, for reliable allele discrimination (Hystad et al., 2015). Despite its structural and allelic diversity, Ppo-D1 exerts a relatively minor effect on grain browning compared with Ppo-A1 (Wang et al., 2009; Nilthong et al., 2012). Quantitative analyses have estimated its contribution to kernel phenotypic variance at approximately 2%, in contrast to the roughly 80% attributed to Ppo-A1, underscoring its secondary role in determining PPO-related phenotypes (Martin et al., 2011).

### 1.7 The Ppo-2 gene: a minor member of the PPO family

The first hypothesis proposing the existence of paralogous genes on chromosomes 2A, 2B, and 2D was advanced by Jukanti et al. (2004), following the identification of six distinct DNA sequences corresponding to Ppo loci. Subsequent studies led to the characterization and formal classification of the genes Ppo-A2, Ppo-B2 and Ppo-D2 (Beecher and Skinner, 2011). These genes were mapped using the Louise x Penawawa mapping population, cloned and sequenced, thereby establishing their chromosomal positions and molecular structures (Beecher et al., 2012).

In bread wheat, eight alleles have been described within the Ppo-2 locus (Table 1; Table 2) (Beecher and Skinner, 2011; Beecher et al., 2012). An additional allele, Ppo-B2d, was later identified in tetraploid wheat, where it occurs in more than 60% of accessions (Taranto et al., 2015). Structurally, Ppo-B2 resembles members of the Ppo-1 locus, consisting of 3 exons and 2 introns, whereas Ppo-A2 and Ppo-D2 comprise two exons and a single conserved intron (Beecher and Skinner, 2011; Beecher et al., 2012; Taranto et al., 2015) (Figure 2).

The coding sequence (CDS) length is 1,673 bp for Ppo-A2 and Ppo-D2, and 1,687 bp for Ppo-B2 (Taranto et al., 2015). Although Ppo-2 genes were initially described as non-kernel genes due to their apparent absence in cDNA libraries derived from grain tissues (Jukanti et al., 2004; Anderson et al., 2006; Fuerst et al., 2008), later studies demonstrated that they are in fact highly expressed during seed development (Beecher and Skinner, 2011). Importantly, the presence or absence of Ppo-B2 transcripts appears to be cultivar-dependent (Beecher and Skinner, 2011; Beecher et al., 2012).

Allelic variation within the Ppo-2 locus has been strongly associated with differences in PPO activity. In bread wheat, Ppo-A2a, Ppo-B2a and Ppo-D2b are associated with high PPO activity, whereas Ppo-A2b, Ppo-B2b, Ppo-B2c and Ppo-D2a are linked to lower activity (Wang et al., 2009; Beecher et al., 2012). In durum wheat, the Ppo-B2d allele has been correlated with higher PPO activity relative to Ppo-B2a (Taranto et al., 2015). Allelic variation at the Ppo-D2 locus influences PPO activity mainly through structural differences affecting gene regulation rather than complete loss of function (Beecher and Skinner, 2011). The Ppo-D2b allele, which retains the second intron and additional upstream regulatory elements, is actively transcribed during early kernel development and contributes substantially to total Ppo transcripts, likely due to enhanced transcription and more efficient mRNA processing or stability. In contrast, Ppo-D2a lacks the second intron, presumably due to an intron loss event, which may impair transcriptional regulation and transcript stability, resulting in lower expression and reduced contribution to PPO activity. Overall, these structural differences modulate enzyme accumulation in developing wheat kernels. Several molecular mechanisms have been proposed to explain these functional differences among alleles. These include the presence of non-conserved missense mutations within or adjacent to catalytic domains that may alter enzyme activity (Beecher et al., 2012); the occurrence of the non-canonical GC–AG splice-sites in the second intron, potentially enabling alternative splicing (Beecher et al., 2012; Taranto et al., 2015); and the presence of functional TFBSs within introns. An example of the latter is the 8-bp insertion containing the INTRONLOWER transcription factor-binding motif in the Ppo-B2d allele (Taranto et al., 2015).

The development of molecular markers has facilitated efficient discrimination of Ppo-2 alleles. Finally, the functional marker MG33 was developed to discriminate the Ppo-B2a allele (555-bp) from Ppo-B2d (549-bp) in durum wheat, with definitive allele identification achieved through sequencing of the amplified products (Taranto et al., 2015).

The precise modulation of PPO activity has become an important objective in modern wheat breeding because PPOs are involved in both enzymatic browning reactions and plant stress responses. Recent advances in molecular genetics and genomics have enabled the development of highly targeted approaches for manipulating Ppo expression while preserving agronomic performance and stress adaptability.

One of the most promising strategies involves tissue-specific promoter engineering (Yaschenko et al., 2022), which allows regulation of Ppo expression in selected tissues or developmental stages. Since PPO enzymes also contribute to defence responses against pathogens and environmental stresses, complete suppression of PPO activity throughout the entire plant may result in undesirable pleiotropic effects (He et al., 2007). By using endosperm-specific or inducible promoters, PPO accumulation can be reduced in grains while maintaining protective PPO activity in vegetative tissues. This approach provides more refined control compared with constitutive silencing strategies.

Genome editing technologies, particularly CRISPR/Cas9 systems, have further revolutionized Ppo manipulation in crops, including wheat (Singha et al., 2022). CRISPR/Cas9 enables precise knockout or modification of target genes such as Ppo-A1 and Ppo-D1 through insertions, deletions, or point mutations. Because bread wheat is a hexaploid species containing multiple homoeologous gene copies, multiplex genome editing (Wang et al., 2014) is especially important for simultaneously targeting several Ppo alleles. Recent studies have demonstrated that CRISPR-mediated editing can significantly reduce PPO activity and improve flour colour without negatively affecting plant growth or yield. Similar genome-editing strategies have already been successfully applied to other quality-related traits in wheat (Sánchez-León et al., 2018; Zhang et al., 2019). Moreover, editing regulatory regions rather than coding sequences may allow fine-tuning of Ppo expression instead of complete gene silencing.

Marker-assisted selection (MAS) represents another efficient strategy for incorporating favourable low-PPO alleles into elite wheat germplasm. Molecular markers tightly linked to Ppo loci enable breeders to identify desirable genotypes at early developmental stages, thereby reducing the need for extensive phenotypic screening (Sun et al., 2005). Marker-assisted pyramiding further extends this concept by combining multiple favourable alleles from different loci into a single genotype (Gautam et al., 2020; Djenadi et al., 2025). The accumulation of low-PPO alleles often produces additive effects that contribute to greater phenotypic stability across environments. Such approaches are particularly valuable in breeding programmes aimed at simultaneously improving technological quality and processing characteristics.

Additional strategies include RNA interference (RNAi), genomic selection, and epigenetic regulation. Genomic selection uses genome-wide marker information to predict PPO-related phenotypes and accelerate breeding cycles, particularly for quantitatively inherited traits.

Overall, the integration of tissue-specific expression systems, CRISPR/Cas9 genome editing, and marker-assisted breeding strategies provides a comprehensive framework for optimizing PPO-associated traits in wheat. Collectively, these technologies support the development of wheat cultivars with improved end-use quality, reduced enzymatic browning, and enhanced market value while preserving resilience to biotic and abiotic stresses.

### 1.8 Unravelling Ppo gene variation through genome-wide association studies

**FIGURE 3:**
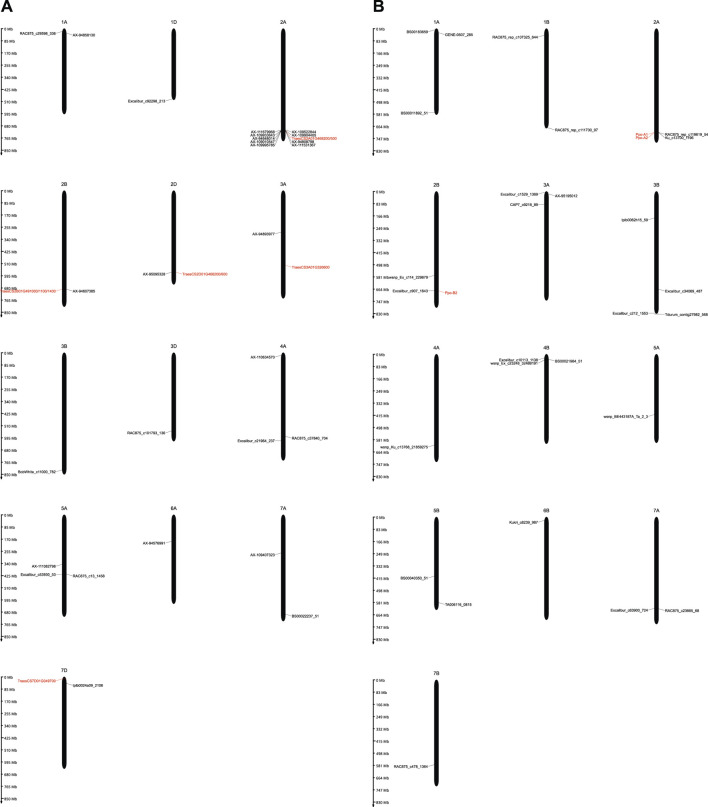
A Physical mapping of Ppo genes and Ppo-associated single nucleotide polymorphism (SNP) in wheat. Ppo genes are shown in red, and SNPs associated with PPO activity, identified through association mapping studies, are shown in black. **(A)** depicts the distribution across the bread wheat (*Triticum aestivum* L.) genome, and **(B)** shows the distribution across the durum wheat (*Triticum turgidum* ssp. *durum*) genome.

Genome-wide association studies (GWAS) have substantially improved the identification of genomic regions associated with PPO activity. Jiang et al. (2018) identified 12 SNP loci on chromosomes 1D, 3D, 4A, 5A, 6A, 7A, and 7D associated with PPO activity, reporting only one stable marker–trait association across two environments, located on chromosome 3D (Figure 3B). Although these regions do not harbour known Ppo genes, signals on chromosomes 3, 5, and 7 were consistent with previously reported QTLs (Zhao et al., 2013; Zhai et al., 2016). Their study also demonstrated that PPO activity is independent of flour colour-related traits, a finding later confirmed by Ji et al. (2021). Additionally, Liu et al. (2020) identified a GWAS peak approximately 123 kb from Ppo-A2 (Figure 3A), suggesting that variation within cis-acting regulatory elements may influence gene expression. More recently, a GWAS on tetraploid wheat by Taranto et al. (2012) significantly advanced the field by identifying several genes in linkage disequilibrium with marker–trait associations, thereby highlighting candidate targets for previously underexplored traits. This analysis pinpointed markers located within or flanking the Ppo genes (Figure 3B) and revealed additional genes involved in biotic and abiotic stress responses (e.g., endochitinases), as well as genes affecting alternative pre-mRNA splicing potentially contributing to variation in gene expression (e.g., Ran-binding zinc finger protein or the splicing factor PRPF18). The study also identified genes related to metallopeptidase activity and metal ion transport, such as copper/iron-binding proteins and the Cu-transporting ATPase RAN1.

### 1.9 Molecular evidence from Ppo genes in wheat evolutionary history

Owing to their structural complexity, Ppo genes have been widely used in phylogenetic studies to trace the evolutionary history of wheats. The clustering of alleles from diploid, tetraploid, and hexaploid wheat species observed by He et al. (2009) supported earlier findings (Dvořák et al., 1993; Ciaffi et al., 2000; Huang et al., 2002; Petersen et al., 2006) that the A genome of both durum and bread wheat originated from *T*. *urartu*. Interestingly, the Ppo-A1e allele of *T. turgidum* ssp. *durum* grouped with the corresponding allele from *T. monococcum*, suggesting either historical genetic exchange between tetraploid and diploid species or the presence of a *T. urartu* accession carrying this allele (He et al., 2009). The Ppo-A1b allele was reported to be shared between *T. aestivum* Chinese spring and a *T. turgidum* ssp. *dicoccoides* line, while Ppo-A1a exhibited high sequence identity with the *T. turgidum* ssp. *dicoccoides* Ppo-A1f allele (He et al., 2009). More recently, Nakamaru et al. (2023) traced the origin of the Ppo-A1i allele in hexaploid wheat to Ppo-A1g, a variant present in some *T. turgidum* ssp. *durum* cultivars such as Svevo. Regarding the D genome, comparative analyses of DNA and protein sequences indicate that Ppo-D1 haplotypes exhibit greater genetic divergence than Ppo-A1 haplotypes (He et al., 2007).

Both He et al. (2007) and He et al. (2009) agree that the observed divergence of Ppo-D1b haplotypes resulted from multiple hybridization events involving more than one *Ae*. *tauschii* accession. Remarkably, Ppo-D1b stands out as the sole allele diverging from both A-genome and other D-genome alleles. Taranto et al. (2015) explored the evolutionary history of *T. turgidum* ssp. *durum* and its B genome, highlighting that modern breeding has preferentially selected alleles with lower activity in Ppo-B1 (Ppo-B1a, Ppo-B1b, and Ppo-B1d). In contrast, 39% of durum wheat cultivars carry the Ppo-B2d allele, which is associated with high PPO activity, suggesting potential for further improvement. Later, Taranto et al. (2021) used PPO activity-related marker-trait associations (MTAs) to investigate genetic divergence among *T. turgidum* subspecies, specifically distinguishing ssp. *carthlicum* from the *durum*, *turanicum*, and *dicoccum* groups. These findings support an evolutionary origin for ssp. *carthlicum* through spontaneous hybridization between *T. aestivum* ssp. *carthlicoides* and wild emmer (Takumi and Morimoto, 2015).

### 1.10 Comprehensive analysis of the PPO gene superfamily atlas in *Triticum aestivum* L.

We used the Wheat Expression Atlas (Supplementary Table S3; https://www.wheat-expression.com/), a high-resolution spatiotemporal resource of the *Triticum aestivum* transcriptome, to investigate the transcriptional landscape of Ppo genes.

By integrating developmental and multi-stress response data, this atlas reveals complex interactions among homoeolog-specific regulation, developmental programming, and environmental responsiveness.

The dataset includes seven primary Ppo genes located on homoeologous group 2: TraesCS2A02G468200 and TraesCS2A02G468500 (A genome); TraesCS2B02G491000, TraesCS2B02G491100, and TraesCS2B02G491400 (B genome); and TraesCS2D02G468200 and TraesCS2D02G468600 (D genome). It spans approximately 55 specialized tissues across more than 50 genotypes, including the reference cultivar Chinese spring, synthetic hexaploids, and Near-Isogenic Lines targeting specific traits. Samples cover a wide range of organs, from roots and leaves to highly resolved reproductive structures such as aleurone, transfer cells, and meiotic stamens, across over 40 developmental stages, from seedling emergence to grain maturation (up to 35 days post-anthesis). In addition, the atlas captures responses to 15 biotic and abiotic stresses, including infections by major fungal pathogens (*Fusarium graminearum*, *Puccinia striiformis*, *Zymoseptoria tritici*) and environmental challenges such as drought, heat, and nutrient deficiency. Its structured metadata enables precise cross-comparison of expression patterns across tissues, developmental stages, and stress conditions.

Overall, Ppo gene expression is highly tissue-specific and developmentally regulated. For instance, TraesCS2D02G468200 shows strong reproductive specialization, with peak expression in spikelets during anthesis (up to 14,415 TPM in the CM variety), whereas TraesCS2A02G468200 is primarily associated with grain maturation, peaking in the seed coat (4,726 TPM in AZHURNAYA). In contrast, most Ppo genes show minimal expression in vegetative tissues, with the notable exception of TraesCS2A02G468500, which is highly induced (>9,000 TPM) in seedlings during immune responses. Stress responsiveness also varies among genes: TraesCS2B02G491100 acts as a biotic stress-responsive gene, while others, such as TraesCS2B02G491400, remain largely unresponsive to certain abiotic stresses.

Although these data highlight highly structured and condition-specific expression patterns within the Ppo gene family, they remain primarily descriptive and should be interpreted cautiously in the absence of dedicated functional validation.

### 1.11 Genetic engineering techniques to understand the role of Ppo genes in browning and response to external stimuli

Advancing our understanding of Ppo gene structure, together with progress in molecular biology, has primarily aimed to develop strategies to mitigate enzymatic browning in wheat-derived products. However, the role of Ppo genes in responses to external stimuli has remained relatively underexplored. In addition, significant challenges have persisted in effectively combining natural allelic variants across different tissues and phenological stages. Advanced biotechnological tools, including RNA interference (RNAi), Targeting Induced Local Lesions IN Genomes (TILLING), and the CRISPR/Cas9 system, have been effectively used to silence or downregulate Ppo genes, thereby reducing enzymatic browning in wheat. RNA interference (RNAi) has also been applied in bread wheat to simultaneously silence homoeologous genes and their paralogues (Travella et al., 2006; Fu et al., 2007). This approach led to a substantial reduction in Ppo-1 transcript levels (15.5%–60.9%) and enzymatic activity (12.9%–20.4%) (Zhai et al., 2023), confirming the predominant contribution of Ppo-1 and suggesting the potential applicability of the same approach to Ppo-2 genes. Additionally, Zhai et al. (2023) elucidated the functional role of the Ppo-1 genes in wheat by employing RNAi and TILLING to reduce Ppo-1 transcript levels by up to 60.9% and PPO activity by nearly 30% in specific mutant lines. A key discovery involved two missense mutants, M092141 and M091098, which harbour mutations at highly conserved glycine residues located at the entrance of the active site’s hydrophobic pocket. These mutations likely restrict substrate access to the enzyme’s catalytic core, thereby markedly reducing overall PPO activity. This work provides a molecular blueprint for the genetic regulation of Ppo-1 and offers valuable germplasm for breeding wheat cultivars with improved colour stability and market potential. Genome editing via the CRISPR/Cas9 system has emerged as a robust strategy for introducing targeted variations across multiple loci simultaneously. In a pioneering study, all three Ppo-1 homoeologues were successfully knocked out in the spring wheat cultivar Fielder leading to a drastic reduction in PPO activity (Zhang et al., 2021). A single guide RNA (sgRNA) was designed to target conserved regions within the third exon of the A, B, and D genomes. While no mutations were detected in the T0 generation, a 2-bp deletion was identified in T1 and stably inherited through to the T3 generation. Analysis of these T3 mutants, which reached a 100% editing ratio, confirmed that the targeted disruption of these loci effectively silenced PPO enzymatic function.

A more extensive study by Wold-McGimsey et al. (2023) modified all copies of Ppo-1 and Ppo-2 (seven genes in total) in three elite cultivars using a sgRNA targeting a conserved copper-binding domain. This strategy achieved strong suppression of PPO activity while enabling precise modification in lines at advanced stages of development (Wold-McGimsey et al., 2023).

Although most biotechnological approaches have focused on Ppo silencing to reduce enzymatic browning, from a defensive perspective Ppo overexpression represented a key component of the plant oxidative shield. In several crops, transgenic lines with elevated PPO levels showed enhanced resistance to a wide range of pathogens and insect pests. For example, increased Ppo expression in tomato conferred resistance to major lepidopteran pests, including *Spodoptera litura*, *Helicoverpa armigera*, and *Spodoptera exigua* (Thipyapong et al., 2007; Bhonwong et al., 2009). Similarly, Ppo upregulation in species such as *Solanum lycopersicum*, *Juglans regia*, and *Fragaria × ananassa* was associated with improved resistance to fungal infections and bacterial pathogens like *Pseudomonas syringae* (Zhang et al., 2021).

The importance of PPOs was further highlighted by the contrasting effects of their suppression. While PPO overexpression in poplar inhibited the growth of *Malacosoma disstria*, plants with reduced Ppo activity, achieved through antisense technology, showed increased susceptibility to herbivory (Wang and Constabel, 2004). Specifically, studies on *Leptinotarsa decemlineata* (Colorado potato beetle) feeding on antisense PPO transgenic tomatoes revealed accelerated larval growth, increased foliage consumption, and higher survival rates compared with those feeding on wild-type or overexpressing counterparts (Mahanil et al., 2008). Although strategies to reduce enzymatic browning were more advanced, reflecting a primary need in food processing, these findings positioned Ppo genes as strong candidates for genetic engineering aimed at enhancing pest and disease resistance in a range of agricultural and forestry species.

### 1.12 PPO control strategies in the food industry

The food industry employs a combination of physical treatments, chemical inhibitors, and controlled processing environments to mitigate PPO-induced browning in wheat, whole wheat flour, and derived products (Harisha et al., 2025). Among physical approaches, thermal treatments have shown measurable effectiveness. For example, microwave processing at 70 °C for 20 min reduces PPO activity by approximately 27.1%, while more intensive conditions (900 W for 80 s) can achieve up to 71.2% inactivation (Yadav et al., 2010). Similarly, steam treatments during wheat tempering reduce PPO activity by about 22% after 90 s of exposure (Poudel and Rose, 2018). However, these methods may also lead to undesirable side effects, including degradation of heat-sensitive nutrients and loss of phenolic compounds, limiting their applicability when product quality must be preserved.

Consequently, increasing attention has shifted toward non-thermal technologies, which enable more selective enzyme inactivation. Supercritical CO_2_ processing and ultrasound-assisted micronization have demonstrated high efficacy; in particular, PPO activity reductions of up to 93% have been reported in wheat bran following micronization to 10 μm combined with high-intensity ultrasound (Habuš et al., 2021). Cold plasma technology represents another promising approach, as it operates under ambient conditions, requires relatively low energy input, and is well suited to dry systems, facilitating industrial scalability (Sruthi et al., 2022). In this context, PPO activity reductions of approximately 49.5% have been achieved in wheat kernels treated at 500 W for 50 s (Li et al., 2022). Moreover, Chakraborty et al. (2025b) demonstrated that combined cold plasma and steam treatments effectively inactivate PPO by altering enzyme structure while preserving the colour and composition of wheat flour.

Overall, these approaches enable substantial enzyme inactivation within short processing times while minimizing adverse effects on nutritional and functional properties. This body of evidence suggests that elevated PPO levels in wheat grain, flour, or end products do not necessarily constitute a limiting constraint, as their impact can be significantly modulated during processing. The extent of PPO reduction achievable through both conventional and emerging technologies indicates that the gap between low- and medium/high-PPO raw materials can be considerably narrowed, often to levels compatible with industrial quality standards.

## 2 Conclusion

PPO enzymes and their corresponding genes have long been central to studies of quality-related traits in wheat. This sustained focus has led to an extensive characterization of their chromosomal localization, gene structure, and allelic diversity, as well as to the development of a comprehensive suite of molecular markers applicable to both bread and durum wheat, as summarized in the present contribution. This body of knowledge has been widely exploited in breeding programmes through marker-assisted selection and, more recently, to directly target Ppo genes and transcripts to suppress their effects. In addition, Ppo-related markers and sequences have been utilized for broader applications, including phylogenetic analyses aimed at reconstructing the evolutionary history of wheat. Despite these advances, several critical knowledge gaps remain that warrant a reassessment of the biological role of PPOs. First, functional studies explicitly demonstrating causal roles of PPOs in biotic and abiotic stress responses are still limited. Future work employing gene knockout, silencing, overexpression, and genome-editing approaches will be essential to determine whether PPOs directly contribute to stress resistance and plant adaptation. Second, the spatiotemporal regulation of PPO expression across tissues and developmental stages remains poorly understood, particularly regarding the coordination between grain-specific PPO activity linked to flour browning and PPO functions in vegetative tissues involved in stress responses. Third, the genetic and regulatory networks interacting with Ppo genes remain largely unexplored. Integrative studies combining transcriptomics, metabolomics, and epistatic analyses will be necessary to identify additional loci, signaling pathways, and gene interactions modulating PPO-related phenotypes. Finally, the environmental regulation of PPO activity and the extent of genotype-by-environment interactions remain insufficiently understood. Future studies should investigate how climatic factors, agronomic practices, and stress conditions modulate Ppo expression and activity across diverse genetic backgrounds, thereby influencing the trade-off between grain quality traits and plant adaptive resilience. In this context, breeding strategies focused solely on reducing PPO-mediated browning to enhance consumer acceptance may represent an oversimplified approach, as they risk overlooking the broader physiological and potentially adaptive roles of PPOs. Collectively, these considerations support a shift away from the long-held “low-PPO is always better” paradigm toward a balanced PPO model, in which PPO activity is fine-tuned to achieve an optimal compromise between plant resilience and flour quality.

## Supplementary material

SUPPLEMENTARY TABLE S1 Structural features of Ppo alleles in wheat.

SUPPLEMENTARY TABLE S2 Molecular markers and primer sequences used for the identification of Ppo allelic variants in wheat.

SUPPLEMENTARY TABLE S3 Transcriptomic metadata and expression profiles (Transcript Per Million; TPM) of Ppo homoeologs in bread wheat, obtained from the Wheat Expression Atlas (accessed 13 January 2026).
